# Potentially inappropriate medications in older Chinese outpatients based on the Beers criteria and Chinese criteria

**DOI:** 10.3389/fphar.2022.991087

**Published:** 2022-09-30

**Authors:** Ying Zhang, Zhaoyan Chen, Fangyuan Tian

**Affiliations:** Department of Pharmacy, West China Hospital, Sichuan University, Chengdu, China

**Keywords:** Elderly patients, polypharmacy, potentially inappropriate medications, Beers criteria, Chinese criteria, outpatient

## Abstract

**Objective:** Polypharmacy increases the prevalence of potentially inappropriate drugs potentially inappropriate medications among older persons, lowering their quality of life. PIMs use can lead to higher mortality in older patients. This study aimed to compare the prevalence of PIMs in older Chinese outpatients according to the Beers criteria and the Chinese criteria and to analyze the risk factors. Second, we describe the differences between the two criteria, focusing on the inappropriate prescription of drugs in older outpatients.

**Methods:** In Chengdu, Southwest China, a cross-sectional study was undertaken using electronic medical data from 9 general hospitals s. Outpatients above the age of 60 who were treated in the Geriatrics Center of these medical institutions were included. The 2019 Beers criteria and the 2017 Chinese criteria were used to evaluate the PIM status of older outpatients, and binary logistic regression was used to identify potential risk factors for PIMs.

**Results:** There were 44,458 prescriptions from 2016 to 2018. The prevalence of PIMs among older outpatients was 30.05% (according to the Beers criteria) and 35.38% (according to the Chinese criteria), with statistical difference. Estazolam, hydrochlorothiazide and alprazolam were the top three PIMs in the Beers criteria, while the top three PIMs in the Chinese criteria were clopidogrel, estazolam and insulin. The prevalence of PIMs was associated with age, the number of diseases and the number of drugs. PIMs were shown to be more common in patients aged 70 and above, with more than 2 kinds of diseases and more than 4 kinds of drugs.

**Conclusion:** PIMs were shown to be common among older outpatients in China, according to this study. The detection rate of the Chinese criteria was higher than that of the Beers criteria.

## Introduction

Comorbidity and polypharmacy in older persons are becoming more common as global aging becomes more serious. Polypharmacy is widespread in older adults, although it is not always avoided. It frequently involves inappropriate medications and can result in negative health effects, such as adverse drug reactions (ADRs), drug–drug interactions or drug-disease interactions (Rieckert et al., 2020). These adverse events often reduce the quality of life of older adults, increase the hospitalization rate and disability rate, and increase the economic burden on the social medical system. (Hamilton et al., 2011; Cahir et al., 2014; Davies and O’Mahony, 2015). Thus, polypharmacy and potentially inappropriate medications (PIMs) become major concerns among older patients.

Potentially inappropriate medication is used when the actual or potential harms of therapy outweigh its benefits. (Lau et al., 2020). Previous studies had showed that the prevalence of PIMs was high, and more adverse drug events, longer hospital stays, more resource use, higher hospital readmission rates and higher health-care expenses were linked to PIMs. (Spinewine et al., 2007; Dalleur et al., 2012; O’Connor et al., 2012; Reich et al., 2014; Hagstrom et al., 2015; Endres et al., 2016; Ma et al., 2018).

Different screening tools are available to assess the level of PIMs in older patients. The most extensively used and cited tools for PIMs were the Beers criteria published in the USA and the STOPP/START criteria published in Europe. The Beers criteria were first published in 1991, and last updated in 2019 (Salbu and Feuer, 2017; The 2019 American Geriatrics Society Beers Criteria ® Update Expert Panel, 2019). The STOPP/START criteria were published in 2008, and updated in 2014 (O'Mahony et al., 2015). They were both explicit criteria, which were used worldwide. In 2017, the Geriatrics Branch of Chinese Medical Association, along with several societies published criteria of potentially inappropriate medications for older adults in China (the Chinese criteria), which were also explicit criteria and widely used in Chinese older patients. (Rational Drug Use Branch of Chinese Association of Geriatric Research, Geriatrics Branch of Chinese Medical Association, Geriatric Medication Committee of Chinese Pharmaceutical Association, Anti-aging and Alzheimer Diseases Committee of Chinese Pharmacological Society, Division of Drug-induced Disease of Chinese Pharmacological Society, 2018).

To date, some studies have investigated the PIMs among Chinese patients based on the Beers criteria or the Chinese criteria, but their studies mainly focused on single hosiptal, or single disease (Ma et al., 2018; Li et al., 2021; Tian et al., 2022). No studies have analyzed the prevalence and risk factors for PIMs in older Chinese outpatients of multiple medical institutions for three consecutive years, and at the same time, reported the differences in the prevalence of PIMs using the 2019 Beers criteria and the 2017 Chinese criteria. Therefore, in this study, we compared the prevalence of PIMs in older Chinese outpatients using the Beers criteria and the Chinese criteria for three consecutive years, investigated relevant risk factors for PIMs, and listed the top drugs discovered using the two sets of criteria. We hope that this study will provide relevant evidence for further research.

## Materials and methods

### Sample and data collection

The prescriptions in this study were from the hospital prescription analytic cooperation project, which was organized by the Chinese Pharmaceutical Association. This project was started in 1997, and was carried out every 3 years. The purpose of the project is to learn about the use of drugs, improve the management of drug use, and enhance the rationality of drug use. The hospitals are invited in this project through project cooperation, and they can share data. The hospitals included in the project were voluntary participants, and provided their prescription data to the project every year. Through the network system, a computer was used to extract prescriptions from hospitals that volunteered to participate in the project. The data extraction rules of the project were that prescriptions of each hospital were randomly selected for 3–4 days every month, and a total of 40 days of prescriptions were randomly selected in 1 year. In Chengdu, nine hospitals volunteered to participate in the project. All nine hospitals were tertiary medical institutions rather than community service centers. The treatment of patients admitted by community service centers is relatively simple. Tertiary medical institutions are comprehensive hospitals, which receive severe patients and use complex drugs, so they are good choices to study PIMs. The project randomly selected prescriptions of all nine hospitals according to the extraction rules. Prescriptions of outpatients aged 60 and above were randomly selected from geriatric departments of the nine hospitals in Chengdu from 1 January 2016, to 31 December 2018. All the information extracted from the computer included the year of prescription, prescription code, drug name, drug specification, administration route, drug price, dosing frequency, dosage, gender, age, and clinical diagnosis. The identities of the hospitals and patients were kept confidential. One prescription had one prescription code, and one prescription code represented one patient. Sample size was calculated by the following formula. $n=\left(z_{a}^{2}\times pq\right)/d^{2}\times 1.5$ A study showed that the prevalence of PIMs in Chinese outpatients was 34.39% (Tian et al., 2021). The sample size of older outpatients in each hospital was calculated to be 275. The total sample size of 9 hospitals should be at least 2475.

### Data inclusion criteria and evaluation criteria

From 1 January 2016 to 31 December 2018, outpatients aged 60 and above in the geriatric department or geriatrics center were included. Two researchers (YZ, FT) independently check prescriptions through an excel. Any inconsistencies between the two researchers were submitted to a third expert, and then resolved through collective discussion. Prescriptions with missing or incomplete information were excluded, such as blank gender, blank age, incomplete diagnosis, blank diagnosis, blank drug, blank dosage, blank administration route, and blank dosing frequency. The prescription was also ruled out if the patient’s gender was uncertain or the patient’s age was inconsistent. When calculating the number of drugs, solvent substances such as water for injection and 0.9% sodium chloride were not included.

### Evaluation of potentially inappropriate medications

The 2019 Beers criteria and the 2017 Chinese criteria were used to evaluate the PIMs of older patients. (The 2019 American Geriatrics Society Beers Criteria ® Update Expert Panel, 2019; Rational Drug Use Branch of Chinese Association of Geriatric Research, Geriatrics Branch of Chinese Medical Association, Geriatric Medication Committee of Chinese Pharmaceutical Association, Anti-aging and Alzheimer Diseases Committee of Chinese Pharmacological Society, Division of Drug-induced Disease of Chinese Pharmacological Society, 2018). The 2019 Beers criteria included potentially inappropriate medication use in older adults, potentially inappropriate medication use in older adults due to drug-disease or drug-syndrome interactions that may exacerbate the disease or syndrome, drugs to be used with caution in older adults, potentially clinically important drug–drug interactions that should be avoided in older adults, and medications that should be avoided or have their dosage reduced with varying levels of kidney function in older adults. (The 2019 American Geriatrics Society Beers Criteria ® Update Expert Panel, 2019). The 2017 Chinese criteria included potentially inappropriate medication use in older adults and potentially inappropriate medication use in older adults in a state of disease or syndrome. (Rational Drug Use Branch of Chinese Association of Geriatric Research, Geriatrics Branch of Chinese Medical Association, Geriatric Medication Committee of Chinese Pharmaceutical Association, Anti-aging and Alzheimer Diseases Committee of Chinese Pharmacological Society, Division of Drug-induced Disease of Chinese Pharmacological Society, 2018). Both criteria focus on the medications of central nervous system, cardiovascular system, endocrine system, gastrointestinal system, and urinary system, as well as anticholinergics, antithrombotics, anti-infective medications and pain medications. However, they also have differences. The Beers criteria also look at proton-pump inhibitors, mineral oils, desmopressin, androgens and drugs that should be avoided or used in reduced doses based on renal function. The Chinese criteria give additional attention to clopidogrel, theophylline, gatifloxacin, vancomycin, clindamycin and PIMs of gout, insomnia, glaucoma, constipation, chronic obstructive pulmonary disease, osteoporosis and hypertension.

Because the patients’ creatinine clearance rates could not be available, we did not assess medications that should be avoided or have their dosage reduced with varying levels of kidney function in older adults. As a result, this study mainly studied the first four groups of the 2019 Beers criteria and the two groups of the 2017 Chinese criteria.

### Statistical analysis

The chi-square test was used to analyze the differences in the characteristics. It was assumed that there was no difference between the compared groups. When the *p*-value was less than 0.05, the hypothesis was not valid, that is, there was a statistical difference between the compared groups. According to the prescription information extracted, sex, age, number of diseases and number of drugs were included in univariate survival analysis. Variables with statistical differences after univariate analysis were included in the binary logistic regression analysis to assess the possible risk factors affecting PIMs in older outpatients. SPSS version 26.0 software was used to perform the statistical analysis.

### Ethics approval

This study protocol was approved by the Sichuan University West China Hospital Research Ethics Board (2020/651). The informed consent is waived by the ethics committee of Sichuan University West China Hospital Research Ethics Board. All procedures performed in this study conformed to the standards of the 1964 Helsinki Declaration and subsequent relevant ethics.

## Results

### Basic characteristics of patients

In this study, all data were extracted into an excel for analysis. One prescription code corresponded to one patient. From 2016 to 2018, a total of 44,857 prescriptions were acquired. Among them, 312 prescriptions with no diagnosis, 77 prescriptions with incomplete diagnosis, 4 prescriptions with missing gender, and 6 prescriptions with only the water for injection were excluded. Finally, 44,458 prescriptions were included. There were 27,358 prescriptions for male patients, representing 61.54% of the total, and 17,100 prescriptions for female patients, accounting for 38.46%. Patients ranged in age from 60 to 119 years old. The number of drugs ranged from 1 to 24, while the number of diseases ranged from 1 to 19. The basic characteristics of the patients are listed in Table 1.

**TABLE 1:** Basic characteristics of patients.

Characteristics	Total(N)	Beers criteria			Chinese criteria		
		Non-PIM(N)	PIM(N)	*p*-value	Non-PIM(N)	PIM(N)	*p*-value
Sex				<0.001			<0.001
Male	27,358	19,402	7,956		17,188	10,170	
Female	17,100	11,698	5,402		11,542	5,558	
Age (Years)				<0.001			<0.001
60–69	15,109	11,515	3,594		11,204	3,905	
70–79	12,958	9,455	3,503		9,006	3,952	
≥80	16,391	10,130	6,261		8,520	7,871	
Number of diseases				<0.001			<0.001
1–2	29,311	21,702	7,609		21,171	8,140	
3–4	8,244	5,532	2,712		4,881	3,363	
≥5	6,903	3,866	3,037		2,678	4,225	
Number of drugs				<0.001			<0.001
1–4	35,147	26,732	8,415		25,964	9,183	
5–9	7,969	4,066	3,903		2,663	5,306	
≥10	1,342	302	1,040		103	1,239	
Total	44,458	31,100	13,358		28,730	15,728	

### Detection of potentially inappropriate medications

According to the Beers criteria, 13,358 (30.05%) of the 44,458 prescriptions had at least one PIM. According to the Chinese criteria, 15,728 prescriptions (35.38%) contained at least one PIM. There was a significant difference in PIM detection between the two groups. The prevalence rates of PIMs are listed in Table 2. Estazolam, hydrochlorothiazide and alprazolam were the top three PIMs in the Beers criteria, while clopidogrel, estazolam and insulin were the top three PIMs in the Chinese criteria. The top ten PIMs are listed in Table 3.

**TABLE 2:** Prevalence of PIMs based on the two criteria.

Year	Total (N)	PIM		
		Beers criteria (N, %)	Chinese criteria (N, %)	*p*-value
2016	15,810	4,343 (27.47%)	5,326 (33.69%)	<0.001
2017	13,128	4,152 (31.63%)	4,822 (36.73%)	<0.001
2018	15,520	4,863 (31.33%)	5,580 (35.95%)	<0.001
Total	44,458	13,358 (30.05%)	15,728 (35.38%)	<0.001

**TABLE 3:** Top ten PIMs based on the two criteria.

Beers criteria (N = 13,358)				
Drugs	Year			Total
	2016 (N = 4,343)	2017 (N = 4,152)	2018 (N = 4,863)	
Estazolam	1,108 (25.51%)	1,260 (30.35%)	1,585 (32.59%)	3,953 (29.59%)
Hydrochlorothiazide	823 (18.95%)	652 (15.70%)	786 (16.16%)	2,261 (16.93%)
Alprazolam	446 (10.27%)	578 (13.92%)	692 (14.23%)	1,716 (12.85%)
Aspirin for primary prevention of cardiovascular disease	366 (8.43%)	300 (7.23%)	286 (5.88%)	952 (7.13%)
Chlorpheniramine	356 (8.20%)	259 (6.24%)	324 (6.66%)	939 (7.03%)
Spironolactone	271 (6.24%)	261 (6.29%)	292 (6.00%)	824 (6.17%)
Glimepiride	386 (8.89%)	275 (6.62%)	97 (1.99%)	758 (5.67%)
Furosemide	190 (4.37%)	180 (4.34%)	216 (4.44%)	586 (4.39%)
Dexzopiclone	85 (1.96%)	137 (3.30%)	183 (3.76%)	405 (3.03%)
Paroxetine	110 (2.53%)	128 (3.08%)	141 (2.90%)	379 (2.84%)
**Chinese criteria (N = 15,728)**				
**Drugs**	**Year**			**Total**
		**2016 (N = 5,326)**	**2017 (N = 4,822)**		**2018 (N = 5,580)**
Clopidogrel	1,741 (32.69%)	1,708 (35.42%)	1,945 (34.86%)	5,394 (34.30%)
Estazolam	1,108 (20.80%)	1,260 (26.13%)	1,585 (28.41%)	3,953 (25.13%)
Insulin	1,145 (21.50%)	799 (16.57%)	841 (15.07%)	2,785 (17.71%)
Alprazolam	446 (8.37%)	578 (11.99%)	692 (12.40%)	1,716 (10.91%)
Theophylline	442 (8.30%)	400 (8.30%)	501 (8.98%)	1,343 (8.54%)
Chlorpheniramine	356 (6.68%)	259 (5.37%)	324 (5.81%)	939 (5.97%)
Nicergoline	164 (3.08%)	165 (3.42%)	139 (2.49%)	468 (2.98%)
Aspirin for renal insufficiency	149 (2.80%)	134 (2.78%)	124 (2.22%)	407 (2.59%)
Diclofenac	73 (1.37%)	107 (2.22%)	79 (1.42%)	259 (1.65%)
Olanzapine	88 (1.65%)	87 (1.80%)	82 (1.47%)	257 (1.63%)

### Factors associated with potentially inappropriate medications

Sex, age, number of diseases and number of drugs were included in univariate analysis, and they were all statistically different. Then, they were included in the binary logistic regression analysis. The reference groups were males, 60–69 years old, 1–2 kinds of diseases and 1–4 kinds of drugs. According to the Beers criteria, gender, age, the number of diseases and the number of drugs all affected the prevalence of PIMs. PIMs were more common in patients who were female (OR 1.359, 95% CI 1.301–1.421), aged 70 and above (OR 1.125, 95% CI 1.065–1.189), had more than 2 kinds of diseases (OR 1.315, 95% CI 1.245–1.389), and had more than 4 kinds of drugs (OR 2.709, 95% CI 2.566–2.859). According to the Chinese criteria, age, the number of diseases and the number of drugs were all linked to an increased risk of PIM use. PIMs were more common in patients aged 70 and above (OR 1.131 95% CI 1.071–1.195), as well as those with more than 2 kinds of diseases (OR 1.656, 95% CI 1.570–1.747) and more than 4 kinds of drugs (OR 4.333, 95% CI 4.101–4.578). Patients who took more than 9 kinds of drugs had the highest risk of PIM use, according to both criteria, as shown in Table 4.

**TABLE 4:** Binary logistic regression analysis for PIMs.

Characteristics	Beers criteria			Chinese criteria		
	OR	95%CI	*p*-value	OR	95%CI	*p*-value
Sex						
Male		References			References	
Female	1.359	1.301–1.421	<0.001	1.017	0.972–1.063	0.471
Age (Years)						
60–69		References			References	
70–79	1.125	1.065–1.189	<0.001	1.131	1.071–1.195	<0.001
≥80	1.411	1.336–1.490	<0.001	1.491	1.412–1.574	<0.001
Number of diseases						
1–2		References			References	
3–4	1.315	1.245–1.389	<0.001	1.656	1.570–1.747	<0.001
≥5	1.238	1.162–1.320	<0.001	1.984	1.860–2.115	<0.001
Number of drugs						
1–4		References			References	
5–9	2.709	2.566–2.859	<0.001	4.333	4.101–4.578	<0.001
≥10	9.295	8.121–10.640	<0.001	23.254	18.935–28.558	<0.001

## Discussion

There are four major findings in our study. First, the Chinese criteria revealed a higher prevalence of PIMs in older Chinese outpatients (35.38%) than the Beers criteria (30.05%). Second, the prevalence of PIMs in older Chinese outpatients gradually increased over three consecutive years, regardless of the Chinese criteria or Beers criteria. Third, Estazolam was the most common PIM according to the Beers criteria, while clopidogrel was the most common PIM according to the Chinese criteria. Finally, Patients aged 70 and above, with more than 2 kinds of diseases, and with more than 4 kinds of drugs were the common risk factors according to two sets of criteria.

Other studies reported the prevalence of PIMs in older outpatients based on the same version of the Beers criteria. Wang et al. investigated 604 hospitalized patients, and the prevalence of PIMs was 55.0%. (Wang et al., 2020). He et al. extracted prescriptions from hospitalized patients for the study, and the prevalence of inappropriate prescriptions was 64.80%. (He et al., 2021). Abdelwahed et al. included 502 older patients, and the PIM prevalence was 34.7%. (Abdelwahed et al., 2021). Achterhof et al. analyzed data of 300 patients, and 53% had at least one PIM. (Achterhof et al., 2020). Li et al. examined 8,235 patients in Chinese communities, and the prevalence of PIMs was 32.16%. (Li et al., 2021). Huang et al. assessed 8477 medications among 1874 patients, and 35.0% of them were PIM-users. (Huang et al., 2020). In our study, the prevalence of PIMs based on the Beers criteria was lower than that based on the Chinese criteria. This might be because the medications in the two criteria differed. In China, 30 of the medications listed in the Beers criteria were not available, as shown in Table 5. Furthermore, the Chinese criteria were biased because they were designed for Chinese patients. At the same time, we found that the prevalence of PIMs in older Chinese outpatients increased gradually from 2016 to 2018, regardless of whether the Chinese criteria or the Beers criteria were used. As a result, enhancing the sensible use of medications in older Chinese outpatients to reduce PIMs remains a major challenge.

**TABLE 5:** Medications from the 2019 Beers criteria not available in China.

Drug category in 2019 Beers criteria	Drugs
First-generation antihistamines	Carbinoxamine
	Dexbrompheniramine
	Doxylamine
	Meclizine
	Pyrilamine
Antispasmodics	Clidinium-chlordiazepoxide
	Dicyclomine
Central alpha-agonists	Guanabenz
	Guanfacine
Antidepressants	Amoxapine
	Desipramine
	Trimipramine
	Nortriptyline
	Protriptyline
Barbiturates	Butabarbital
	Butalbital
	Mephobarbital
	Pentobarbital
Benzodiazepines	Quazepam
Ergoloid mesylates	Isoxsuprine
Skeletal muscle relaxants	Carisoprodol
	Cyclobenzaprine
	Metaxalone
	Orphenadrine
Antipsychotics	Pimavanserin
Antimuscarinics (urinary incontinence)	Darifenacin
	Fesoterodine
Others	Mineral oil
	Prasugrel
	Dextromethorphan/quinidine

Estazolam, hydrochlorothiazide and alprazolam were the top three PIMs in the Beers criteria, while clopidogrel, estazolam and insulin were the top three PIMs in the Chinese criteria. The difference in results was primarily attributable to the disparity between the two criteria. Diuretics were included in the Beers criteria as drugs to be used with caution in older adults due to the risk of hyponatremia, and regular monitoring of sodium was recommended when starting or altering dosages in older adults. (Hix et al., 2011; Correia et al., 2014; Filippatos et al., 2017; Grattagliano et al., 2018; Zhang and Li, 2020). Diuretics were excluded from the Chinese criteria; however, clopidogrel was included because of the hematologic and neurological adverse reactions associated with clopidogrel. (Zakarija et al., 2004; Tiaden et al., 2005; Balamuthusamy and Arora, 2007; Pflumm et al., 2008; Zakarija et al., 2009). On the other hand, the Beers criteria did not include clopidogrel. Despite the fact that insulin was included in both criteria, there were still discrepancies. The Beers criteria only included short- or rapid-acting insulins that were not used in conjunction with basal or long-acting insulins, while the Chinese criteria included all insulins. This led to a dramatic increase in the detection of insulins as PIMs.

Although the detection of insulin differed between the two criteria, both of them included insulin as PIM due to higher risk of hypoglycemia without improvement in hyperglycemia management. However, it is important to note that insulin is also necessary in some older patients with diabetes. In older patients with type 1 diabetes, insulin therapy is necessary. In addition, insulin treatment is also needed in older patients with type 2 diabetes, who fail to achieve glycemic target after lifestyle intervention and non-insulin therapy. To reduce the risk of hypoglycemia in older patients, we can do as follows: 1) Before starting insulin, the benefit of insulin therapy and the risk of hypoglycemia in older diabetic patients should be fully considered, and individualized treatment regimen should be developed. 2) When starting insulin therapy, basal insulins with stable serum concentration are preferred (American Diabetes Association, 2021a). 3) Older patients who are taking insulin should be evaluated whether insulin therapy is necessary or can be simplified. For older patients whose blood glucose can be controlled by non-insulin therapy, insulin should be gradually reduced and stopped. Besides, reducing injection frequency and adding short-acting insulin when the postprandial blood glucose is not satisfied after the use of long-acting insulin should be considered in older patients who need insulin to achieve satisfactory blood glucose control (LeRoith et al., 2019; American Diabetes Association, 2021b).

Estazolam and alprazolam, both benzodiazepines, were highly frequent PIMs according to the Beers criteria and the Chinese criteria. Some studies have shown that benzodiazepines are commonly used in older adults, and there is a long-term use phenomenon. (Kurko et al., 2015; Olfson et al., 2015; Maust et al., 2016). Benzodiazepines can lead to serious injuries such as falls, fractures, cognitive impairment and car accidents, putting a strain on society’s finances and judicial system. (Madhusoodanan and Bogunovic, 2004; Schroeck et al., 2016). Older adults are at risk from long-term benzodiazepine use. In addition, studies have shown that benzodiazepines are linked to the development of dementia or cognitive impairment; (Crowe and Stranks, 2018; Lucchetta et al., 2018; Picton et al., 2018); thus, patients with dementia or cognitive impairment should avoid taking them. Regarding the risks associated with benzodiazepines in older adults, several alternative therapies have been proposed to reduce their use. (Markota et al., 2016).

Potentially independent factors associated with PIMs were identified as patients aged 70 and above, with more than 2 kinds of diseases, and with more than 4 kinds of drugs (*p* < 0.001). Patients who took more than 9 kinds of drugs had the highest risk of PIM use. Polypharmacy was linked to not only PIMs but also medication compliance. Age, medication classes and medication knowledge were found to be associated with medication compliance in a Chinese study of older adults with polypharmacy. (He et al., 2021).

Now, several tools for PIMs identification have been developed to help doctors and pharmacists reduce PIMs. PIM dashboard is a clinical decision-making tool embedded in medical system. It can identify patients who use PIMs and show the type and number of PIMs (Zullo et al., 2018; Richter Lagha et al., 2020). Electronic medical record (EMR) alerts is a system of pop-up alerts in EMRs. It can inform PIMs, adverse drug reactions and drug interactions (Alagiakrishnan et al., 2019). MedStopper webpage is a tool for frail older patients. It can assess PIMs and drug overuse (Cassels, 2017).

In addition to the above tools, multidisciplinary medication makes a contribution to reducing PIMs (Krause, 2019). Clinical pharmacist is an important member of the multidisciplinary team. Clinical pharmacists carry out drug reorganization, drug counseling and pharmaceutical care, find inappropriate medication, give professional advices on patient treatment, reduce avoidable adverse drug reactions and adverse interactions. As a full member, clinical pharmacists can reduce PIMs in elderly patients by 20% or even more (Stuhec et al., 2021; Stuhec and Lah, 2021; Stuhec, 2021).

Several limitations of this study should be noted. First, this study was a retrospective study including a specific region of China, so the results could not be applied to other countries. Second, this study only included outpatients, which could not reflect the characteristics of hospitalized patients. Third, this study only included older patients in geriatric departments, so it could not represent patients in other departments. Next, patients’ creatinine clearance rates could not be available, and this limited us assess medications that should be avoided or have their dosage reduced with varying levels of kidney function in older adults. Besides, prescriptions were only extracted for 40 days in 1 year, which did not represent the whole year. Finally, the adverse outcomes caused by PIMs were not available, so we could not determine which criteria were more suitable for the evaluation of PIMs.

## Conclusion

This study showed that the prevalence of PIMs according to the Chinese criteria was higher than that according to the Beers criteria, and there was a statistical difference between them. The prevalence of PIMs in older Chinese outpatients gradually increased from 2016 to 2018, and benzodiazepines were the most common PIMs. In view of this, we suggest that doctors and pharmacists cooperate to identify the indications of drugs, improve patient compliance, and reduce PIMs in older adults.

## Data Availability

The original contributions presented in the study are included in the article/supplementary material, further inquiries can be directed to the corresponding author.
