# Supplementary material for: Potentially inappropriate medications in older Chinese outpatients based on the Beers criteria and Chinese criteria
Source: Front Pharmacol. 2022 Sep 30;13:991087. doi: 10.3389/fphar.2022.991087 (PMC9561887; doi:10.3389/fphar.2022.991087)
Supplement: Supplementary file 1 [file Table1.DOCX]

**PIMs based on Beers criteria and Chinese criteria**

**S1 Table. PIMs based on Beers criteria**

| **Potentially Inappropriate Medication Use in Older Adults** | | | | | |
| --- | --- | --- | --- | --- | --- |
| Drugs | | 2016 | 2017 | 2018 | Total |
| First-generation antihistamines: Chlorpheniramine, Diphenhydramine (oral), Promethazine, Triprolidine, Brompheniramine | | 464 | 346 | 416 | 1226 |
| Antiparkinsonian agents: Trihexyphenidyl | | 1 | 2 | 2 | 5 |
| Antispasmodics: Atropine (excludes ophthalmic), Belladonna alkaloids, Hyoscyamine | | 58 | 4 | 1 | 63 |
| Dipyridamole, oral short-acting(does not apply to the extendedrelease combination with aspirin) | | 0 | 0 | 2 | 2 |
| Peripheral alpha-1 blockers: Doxazosin, Prazosin, Terazosin | | 35 | 26 | 38 | 99 |
| Central alpha blockers: Reserpine (>0.1 mg/d) | | 1 | 0 | 0 | 1 |
| Digoxin | | 18 | 8 | 14 | 40 |
| Antidepressants, alone or in combination: Amitriptyline, Doxepin (>6 mg/d), Paroxetine | | 177 | 188 | 203 | 568 |
| Antipsychotics, first- (conventional) and second- (atypical) generation: Chlorpromazine, Tiapride, Flupentixol, Aripiprazole, Olanzapine, Quetiapine, Risperidone | | 137 | 174 | 198 | 509 |
| Barbiturates: Phenobarbital | | 0 | 2 | 0 | 2 |
| Benzodiazepines (Short- and intermediate- acting): Alprazolam, Estazolam, Lorazepam | | 1558 | 1838 | 2278 | 5674 |
| Benzodiazepines (Long-acting): Chlordiazepoxide, Clonazepam, Diazepam | | 101 | 85 | 100 | 286 |
| Nonbenzodiazepine, benzodiazepine receptor agonist hypnotics: Eszopiclone, Zolpidem, Zaleplon | | 145 | 213 | 265 | 623 |
| Androgens: Testosterone | | 0 | 2 | 0 | 2 |
| Estrogens with or without progestins: Ethinylestradiol and Cyproterone | | 0 | 0 | 2 | 2 |
| Insulin | | 60 | 54 | 38 | 152 |
| Megestrol | | 22 | 23 | 15 | 60 |
| Sulfonylureas, long acting: Glimepiride, Glibenclamide | | 388 | 275 | 97 | 760 |
| Metoclopramide | | 1 | 1 | 1 | 3 |
| Meperidine | | 1 | 1 | 0 | 2 |
| Indomethacin | | 13 | 16 | 15 | 44 |
| Skeletal muscle relaxants: Chlorzoxazone | | 11 | 12 | 8 | 31 |
| Desmopressin | | 0 | 0 | 1 | 1 |
| **Potentially Inappropriate Medication Use in Older Adults Due to Drug–Disease or Drug–Syndrome Interactions That May Exacerbate the Disease or Syndrome** | | | | | |
| Disease or Syndrome | Drugs | 2016 | 2017 | 2018 | Total |
| Heart failure | NSAIDs and COX-2 inhibitors (Aspirin, Ibuprofen, Paracetamol, Loxoprofen, Pranoprofen, Celecoxib, Diclofenac, Indomethacin); Nondihydropyridine CCBs (Diltiazem); Cilostazol | 57 | 45 | 70 | 172 |
| Delirium | Estazolam | 0 | 0 | 1 | 1 |
| Dementia or cognitive impairment | Anticholinergics (Chlorpheniramine, Trihexyphenidyl, Amitriptyline, Paroxetine, Tolterodine, Solifenacin, Brompheniramine); Benzodiazepines (Alprazolam, Estazolam, Clonazepam); Nonbenzodiazepine, benzodiazepine receptor agonist hypnotics (Eszopiclone, Zolpidem); Antipsychotics (Olanzapine, Quetiapine, Aripiprazole, Flupentixol, Risperidone) | 79 | 69 | 91 | 239 |
| History of falls or fractures | Antipsychotics (Flupentixol, Olanzapine); Benzodiazepines (Alprazolam, Estazolam, Clonazepam); Nonbenzodiazepine, benzodiazepine receptor agonist hypnotics (Eszopiclone, Zolpidem); SSRIs (Escitalopram, Paroxetine, Sertraline); Opioids (Codeine, Oxycodone, Tramadol) | 34 | 24 | 34 | 92 |
| Parkinson disease | Antipsychotics (Olanzapine, Flupentixol, Risperidone) | 7 | 1 | 5 | 13 |
| Chronic kidney disease Stages IV or less (creatinine clearance <30 mL/min) | NSAIDs (Aspirin, Pranoprofen) | 4 | 6 | 6 | 16 |
| Lower urinary tract symptoms, benign prostatic hyperplasia | Strongly anticholinergic drugs, except antimuscarinics for urinary incontinence (Chlorpheniramine, Amitriptyline, Doxepin>6 mg/d, Paroxetine, Olanzapine, Belladonna, Brompheniramine, Triprolidine) | 95 | 63 | 43 | 201 |
| **Potentially Inappropriate Medications to Be Used with Caution in Older Adults** | | | | | |
| Drugs | | 2016 | 2107 | 2018 | Total |
| Aspirin for primary prevention of cardiac events | | 366 | 300 | 286 | 952 |
| Dabigatran, Rivaroxaban | | 8 | 15 | 51 | 74 |
| Antipsychotics, Diuretics, Carbamazepine, Mirtazapine, Oxcarbazepine, SNRIs, SSRIs, TCAs, Tramadol | | 1983 | 1914 | 2247 | 6144 |
| **Potentially Clinically Important Non-Anti-infective Drug–Drug Interactions That Should Be Avoided in Older Adults** | | | | | |
| Drug–Drug Interactions | | 2016 | 2017 | 2018 | 合计 |
| Opioids + Benzodiazepines | | 25 | 59 | 59 | 143 |
| Opioids + Gabapentin | | 0 | 0 | 2 | 2 |
| Anticholinergic + Anticholinergic | | 39 | 31 | 22 | 92 |
| Antidepressants (TCAs, SSRIs, and SNRIs) Antipsychotics Antiepileptics Benzodiazepines and nonbenzodiazepine, benzodiazepine receptor agonist hypnotics Opioids Any combination of three or more of these CNS-active drugs | | 31 | 40 | 45 | 116 |
| Corticosteroids, oral or parenteral + NSAIDs | | 4 | 10 | 13 | 27 |
| Peripheral Alpha-1 blockers + Loop diuretics | | 0 | 1 | 2 | 3 |
| Warfarin + Amiodarone | | 0 | 2 | 5 | 7 |
| Warfarin + NSAIDs | | 1 | 5 | 3 | 9 |

**S2 Table. PIMs based on Chinese criteria**

| **Potentially Inappropriate Medication Use in Older Adults** | | | | | |
| --- | --- | --- | --- | --- | --- |
| Drugs | | 2016 | 2017 | 2018 | Total |
| Lorazepam | | 4 | 0 | 1 | 5 |
| Alprazolam | | 446 | 578 | 692 | 1716 |
| Trihexyphenidyl | | 1 | 2 | 2 | 5 |
| Estazolam | | 1108 | 1260 | 1585 | 3953 |
| Nicergoline | | 164 | 165 | 139 | 468 |
| Zolpidem | | 59 | 76 | 82 | 217 |
| Fluoxetine | | 32 | 17 | 18 | 67 |
| Risperidone | | 4 | 5 | 2 | 11 |
| Olanzapine | | 88 | 87 | 82 | 257 |
| Quetiapine | | 5 | 10 | 5 | 20 |
| Diclofenac | | 73 | 107 | 79 | 259 |
| Ibuprofen | | 38 | 38 | 29 | 105 |
| Reserpine (＞0.1mg/d) | | 1 | 0 | 0 | 1 |
| Doxazosin | | 26 | 39 | 29 | 94 |
| Digoxin (＞0.125mg/d) | | 18 | 8 | 14 | 40 |
| Amiodarone | | 18 | 16 | 17 | 51 |
| Chlorpheniramine | | 356 | 259 | 324 | 939 |
| Insulin | | 1145 | 799 | 841 | 2785 |
| Warfarin | | 69 | 66 | 97 | 232 |
| Clopidogrel | | 1741 | 1708 | 1945 | 5394 |
| Spironolactone (＞25mg/d) | | 57 | 47 | 47 | 151 |
| Theophylline | | 442 | 400 | 501 | 1343 |
| Chlordiazepoxide | | 1 | 0 | 0 | 1 |
| Phenobarbital | | 0 | 2 | 0 | 2 |
| Clonazepam | | 86 | 78 | 91 | 255 |
| Diazepam | | 14 | 7 | 9 | 30 |
| Phenytoin | | 2 | 3 | 2 | 7 |
| Amitriptyline | | 60 | 58 | 60 | 178 |
| Chlorpromazine | | 1 | 1 | 0 | 2 |
| Doxepin | | 7 | 2 | 2 | 11 |
| Haloperidol | | 1 | 0 | 0 | 1 |
| Aripiprazole | | 0 | 3 | 7 | 10 |
| Indomethacin | | 13 | 16 | 15 | 44 |
| ≥2 NSAIDs | | 98 | 71 | 59 | 228 |
| Naproxen | | 4 | 1 | 3 | 8 |
| Ketoprofen | | 2 | 2 | 1 | 5 |
| Etoricoxib | | 1 | 2 | 11 | 14 |
| Gatifloxacin | | 1 | 0 | 0 | 1 |
| Aminoglycoside antibiotics | | 29 | 38 | 32 | 99 |
| Clindamycin | | 1 | 0 | 0 | 1 |
| Promethazine | | 3 | 2 | 2 | 7 |
| Diphenhydramine | | 2 | 0 | 0 | 2 |
| Glibenclamide | | 2 | 0 | 0 | 2 |
| Megestrol | | 22 | 23 | 15 | 60 |
| Hyoscyamine | | 52 | 0 | 0 | 52 |
| Belladonna alkaloids | | 6 | 4 | 1 | 11 |
| Meperidine | | 1 | 1 | 0 | 2 |
| Morphine | | 5 | 11 | 1 | 17 |
| Tramadol | | 25 | 28 | 42 | 95 |
| Baclofen | | 0 | 0 | 3 | 3 |
| Chlorzoxazone | | 11 | 12 | 8 | 31 |
| Tolterodine | | 28 | 20 | 11 | 59 |
| **Potentially Inappropriate Medication Use in Older Adults with Specific Diseases** | | | | | |
| Disease | Drugs | 2016 | 2017 | 2018 | Total |
| Seizures or epilepsy | Olanzapine, Quetiapine | 2 | 0 | 1 | 3 |
| Delirium | Estazolam | 0 | 0 | 1 | 1 |
| Dementia or cognitive impairment | Alprazolam, Estazolam, Clonazepam | 34 | 36 | 39 | 109 |
| Parkinson disease | Olanzapine, Flupentixol, Risperidone | 7 | 1 | 5 | 13 |
| Cognitive impairment | Chlorpheniramine, Brompheniramine, Trihexyphenidyl, Amitriptyline, Paroxetine, Tolterodine, Solifenacin, Olanzapine | 37 | 28 | 42 | 107 |
| Heart failure | Aspirin, Ibuprofen, Paracetamol, Loxoprofen, Pranoprofen, Celecoxib, Diclofenac, Indomethacin, Diltiazem, Cilostazol | 57 | 45 | 70 | 172 |
| Hypertension | Ibuprofen, Aceclofenac, Loxoprofen, Meloxicam, Naproxen, Nimesulide, Pranoprofen, Celecoxib, Diclofenac, Ketoprofen, Etofenamate, Etoricoxib, Indomethacin, Reserpine | 103 | 126 | 101 | 330 |
| Coagulation disorders or anticoagulant therapy | Clopidogrel, Aspirin, Diclofenac, Meloxicam, Imrecoxib, Etoricoxib | 5 | 11 | 12 | 28 |
| Renal insufficiency | Aspirin, Paracetamol, Ibuprofen, Aceclofenac, Loxoprofen, Meloxicam, Indomethacin, Pranoprofen, Celecoxib, Diclofenac, Etoricoxib | 251 | 196 | 191 | 638 |
| Lower urinary tract symptoms, benign prostatic hyperplasia | Chlorpheniramine, Amitriptyline, Doxepin, Paroxetine, Olanzapine, Belladonna, Brompheniramine, Triprolidine | 95 | 63 | 43 | 201 |
| Peptic ulcer | Prednisone | 0 | 1 | 3 | 4 |
| Chronic obstructive pulmonary disease | Alprazolam, Estazolam, Lorazepam, Clonazepam | 62 | 116 | 176 | 354 |
| Osteoporosis | Dexamethasone, Betamethasone, Methylprednisolone, Prednisone | 7 | 8 | 15 | 30 |
| Diabetes | Methylprednisolone | 3 | 2 | 4 | 9 |
| History of falls or fractures | Alprazolam, Estazolam, Clonazepam, Olanzapine, Flupentixol, Eszopiclone | 21 | 21 | 29 | 71 |
| Glaucoma | Chlorpheniramine, Brompheniramine | 4 | 2 | 0 | 6 |
| Gout | Hydrochlorothiazide, Indapamide | 5 | 3 | 5 | 13 |
